# A Helping Hand during *t*-Testing Times

**DOI:** 10.1371/journal.pbio.1001785

**Published:** 2014-02-18

**Authors:** Siouxsie Wiles

**Affiliations:** 1 Bioluminescent Superbugs Lab, Department of Molecular Medicine and Pathology, University of Auckland, New Zealand; 2 Maurice Wilkins Centre for Molecular Biodiscovery, Auckland, New Zealand

## Abstract

Siouxsie Wiles reviews *The Cartoon Introduction to Statistics* by cartoonist Grady Klein and statistician Alan Dabney.

[Fig pbio-1001785-g001]I vividly remember the lecturer who attempted to teach me statistics as an undergraduate. The poor man tasked with imparting such vital knowledge alas had no ability to engage a large class of budding biologists. Each lecture he mumbled his way through his notes, writing out long, incomprehensible equations on the board, as paper aeroplanes bounced off his back. Talking to other biologists, it is clear that my experience wasn't unique.

**Figure pbio-1001785-g001:**
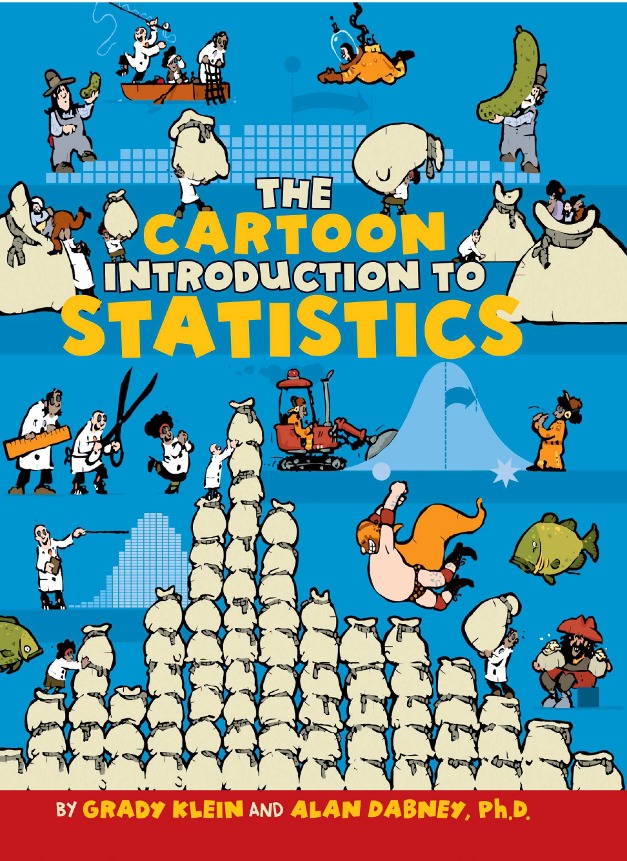
Klein G, Dabney A (2013) The Cartoon Introduction to Statistics. New York: Hill and Wang. 240 pp. ISBN 978-0809033591 (paperback).

With training like this, perhaps it's no surprise to hear that around half of published biomedical papers are thought to contain mistakes in data presentation and statistical analysis [1]–[3]. Mistakes include failing to account for multiple comparisons, which can lead to false-positive results, and using tests that assume a normal distribution on data that are skewed. Assumptions of normality are likely to be particularly prevalent when it comes to the analysis of small datasets, such as those generated during research projects involving animals. Such experiments are kept small (but not too small) for ethical reasons, and as such often cannot even be mathematically tested for normality [4].

This fundamental failure to properly address how to best teach statistics and experimental design to biologists has left our community with several generations of principal investigators, journal reviewers, and editors unaware that there is even a problem. Further, the problem is self-perpetuating, as badly presented and incorrectly analysed data often get published in high impact journals, and the methodology emulated by other authors. Perhaps this is how we have arrived at the current situation, where many biologists believe that one-size-fits-all when it comes to performing statistics, the “one size” almost always being the Student's *t*-test.

With the biological sciences moving rapidly into the “omics/big data” era, which has an inherent need for relatively sophisticated data analysis techniques, we may find that a more maths-savvy generation of biologists arises. In the meantime though, how can we address this issue? A great start would be to make every biologist read *The Cartoon Introduction to Statistics* by cartoonist Grady Klein and statistician Associate Professor Alan Dabney (Texas A&M University). In their excellent and engaging book, a cast of pirates, aliens, rhinos, flying pigs, and dragon-riding Vikings explain a range of important concepts including bias, means, medians, variation, and hypothesis testing. Like me, Klein and Dabney see no reason to show biologists pages and pages of mathematical formulae to explain these concepts. Instead, Klein has conjured up a “stats machine” that would make Wallace and Gromit proud (“You put your random numbers in here … slide this thingy over … and out pops a *p*-value!”), and Dabney leaves all the technical details for “The Math Cave” at the end of the book.

If nothing else, I would hope that biologists reading Klein and Dabney's book take home just one message: to really look at their data; how did they gather it, how much do they have of it, and how is it distributed? In all honesty, this book won't solve the problem of just how biologists choose the correct statistical test for their data, but hopefully it will pique their interest sufficiently to find out for themselves. As for the wider issue of improving statistical literacy in the biological sciences, it is clear that a range of approaches are currently needed. Lecturers could do to take a leaf out of Klein and Dabney's book and ditch the maths when teaching biologists. Alongside this should be the requirement for engaging stats courses for all post-graduate students carrying out research.

As for principal investigators and journal editors? Perhaps the simplest approach would be to provide clear guidance about what tests are relevant for which kind of data. Indeed, there are a number of easy to follow resources and decision charts available which could be used to either determine the appropriate test to carry out, or in the case of reviewers and editors, from which they could work backwards to see whether a particular test was used appropriately [5]–[9].

Nowadays, performing a statistical test merely requires clicking a few buttons in a computer programme. By ensuring biologists are provided with guidance about which buttons to click, and why, I strongly believe that we can raise the standard of data presentation and statistical analysis so that published papers accurately represent the data they describe.

**About the Author**

Dr. Siouxsie Wiles is an award-winning microbiologist and bioluminescence enthusiast who heads up the Bioluminescent Superbugs Lab at the University of Auckland in New Zealand. Here she combines her twin passions to understand and combat infectious diseases. In a nutshell, Siouxsie and her team make nasty bacteria glow in the dark. Further information about Dr. Wiles' research can be found on her website at http://superbugslab.org.
